# Visceral Leishmaniasis in West Africa: Clinical Characteristics, Vectors, and Reservoirs

**DOI:** 10.1155/2019/9282690

**Published:** 2019-09-02

**Authors:** Abdoulaye K. Kone, Doumbo Safiatou Niaré, Martine Piarroux, Arezki Izri, Pierre Marty, Matthew B. Laurens, Renaud Piarroux, Mahamadou A. Thera, Ogobara K. Doumbo

**Affiliations:** ^1^Malaria Research and Training Center, Department of Epidemiology of Parasitic Diseases, Faculty of Medicine, and Dentistry, UMI-3189, University of Science, Technique and Technology of Bamako, BP 1805, Bamako, Mali; ^2^Institut Pierre Louis d'Épidémiologie et de Santé Publique, INSERM UMR S1136, Sorbonne University, Paris, France; ^3^Parasitology-Mycology, Hôpital Avicenne, Paris 13 University, UMR 190, Aix-Marseille University, France; ^4^Inserm U1065, Centre Méditerranéen de Médecine Moléculaire, C3M, Université de Nice-Sophia Antipolis, 151, route St Antoine de Ginestière, BP 2 3194, 06204 Nice Cedex, France; ^5^Parasitologie-Mycologie, Hôpital de l'Archet, Centre Hospitalier Universitaire de Nice, France; ^6^Center for Vaccine Development and Global Health, University of Maryland School of Medicine, 685 W Baltimore St, Rm 480, Baltimore, Maryland, USA

## Abstract

Visceral leishmaniasis (VL) is the most serious form of human leishmaniasis. VL is understudied in West Africa. The increasing number of patients at-risk, including persons living with HIV and other chronic immunosuppressive diseases, and likely underreporting of VL related to diagnostic challenges advocate for review of existing data to understand VL regional epidemiology. Our review aims to describe the clinical characteristics and epidemiology of Human VL (HVL) in West Africa. We conducted a literature search to identify peer-reviewed articles and grey literature sources using the search terms “Visceral leishmaniasis West Africa”, “*Leishmania donovani *West Africa”; and “*Leishmania infantum *West Africa”. Thirty published articles report HVL from seven countries, including The Gambia, Niger, Nigeria, Ivory Coast, Togo, Burkina Faso, and Guinea Bissau. Three countries report cases of Canine Visceral Leishmaniasis (CVL), including The Gambia, Senegal, and Burkina Faso. Niger, Nigeria, and Ivory Coast report the greatest number of HVL cases. As VL is present in West Africa, active surveillance, increased diagnostic capacity, and studies of vectors and reservoirs are essential to better understand VL epidemiology in the region.

## 1. Introduction

VL is caused by a flagellated protozoan of the genus* Leishmania spp.* (Trypanosomatida, Trypanosomatidae) and is transmitted by the infective bite of female sandflies (Diptera, Psychodidae) of the genus* Phlebotomus* in the Old World and* Lutzomyia* in the New World. An estimated 50,000 to 90,000 new cases of VL occur worldwide every year, causing fatality in over 95% of cases if left untreated [1]. From 2004 to 2008, 56,700 cases of HVL have been reported in East Africa, but few cases have been reported in West Africa [2]. According to the World Health Organization (WHO) report 2017, four of the six West African countries not providing data are endemic for VL, Ivory Coast, Niger, Mauritania, and Senegal, and the ten remaining nations are free from VL, but most nations in the region did not provide data on VL in 2016 (see Figure 2). From 2005 to 2017, 60 cases of VL were reported from West African nations to WHO, and 57 of these cases occurred in Nigeria in 2012 [3]. Two species of* Leishmania* implicated in HVL are found in West Africa:* Leishmania donovani*, which is anthroponotic and was incriminated in an HVL outbreak [4] and* Leishmania infantum*, mostly found in dogs [5, 6], which is responsible for sporadic cases in humans. The first case of HVL caused by* Leishmania infantum* in West Africa was reported from The Gambia [7] and later from other West African countries [8, 9]. HVL has also been reported from the Ivory Coast and Niger; cases were confirmed by microscopy without* Leishmania* species identification [10–12].

The clinical features, vectors, and reservoirs of VL in West Africa are not well known. In this review, we aim to describe clinical and epidemiological profiles of VL in West Africa.

## 2. Materials and Methods

We conducted a literature search to identify records of VL in West Africa. The search included peer-reviewed manuscripts in both French and English using HINARI, Google Scholar, and PubMed, with the following terms: “Visceral leishmaniasis West Africa”; “*Leishmania donovani *West Africa”; and “*Leishmania infantum *West Africa”. Unpublished data on VL were searched on Google using the same search terms.

We conducted an exhaustive coverage strategy to find published and unpublished papers on VL in West Africa. The articles were selected by the first author if relevant to review subject. When full text was not available, the abstract was used. The coauthors verified these selected full text articles and abstracts for relevance. The data were extracted and grouped by clinical cases, reservoirs, and vectors.

The following data were extracted from each study: study location (country), patient population, clinical and laboratory characteristics at presentation, diagnosis tests performed, vectors (description of fauna), and potential parasite reservoirs.

## 3. Results

Thirty relevant peer-reviewed manuscripts were found. Most original articles describing HVL dated from the early 2000's and before, but articles describing reservoirs were between 2011 and 2017.

**Figure 1:**
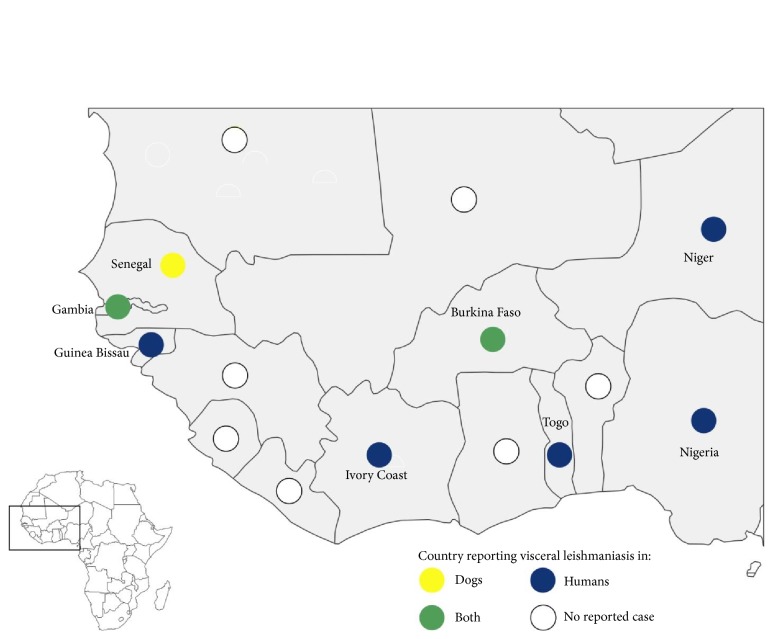
Geographic distribution of CVL and HVL in West Africa 1949-2018 (made with Philcarto, http://philcarto.free.fr/index.html).

Cases of HVL and CVL were distributed all over West Africa (see Figure 1).

Based on these records, two countries, Niger and Ivory Coast, reported the greatest number of HVL cases confirmed by microscopy or serology. Niger reported 21 cases, Ivory Coast reported eight cases, and the Gambia and Burkina Faso reported one case each. Most of these cases occurred more than ten years ago: one Togolese case was diagnosed in Lama-Kara (Northern region) in 1994; and in Nigeria suspected cases have been reported between 1936 and 1947 [14] and 60 cases between 2005 and 2012 [3]. The Gambian cases were reported in 1949, 1980, and 1982 near Fajara [7, 15, 16]. A case of VL in a European boy in Upper Volta (now called Burkina Faso) was described in 1978 [17].* Leishmania* species were not specified for these cases. In Republic of Guinea, serological surveys in humans showed positive reactions to* L. donovani sensu lato* antigen, but no symptomatic VL was described [18] (see Table 1).

## 4. Discussion

### 4.1. Epidemiology

HVL cases reported in West African are rare compared to other African endemic regions such as Eastern Africa. The status of* L. donovani* VL in Eastern Africa is quite different and characterized by an increased number of cases in the past decade, due to ongoing armed conflicts that leave people without housing and health infrastructure, with increased exposure to sandflies bites, and make it difficult to manage cases and deliver vector control activities. These armed conflicts caused massive movements of susceptible or infected populations, respectively, into VL-endemic or nonendemic areas, with widespread malnutrition and famine contributing to increased risk of VL and epidemics [19].

**Figure 2:**
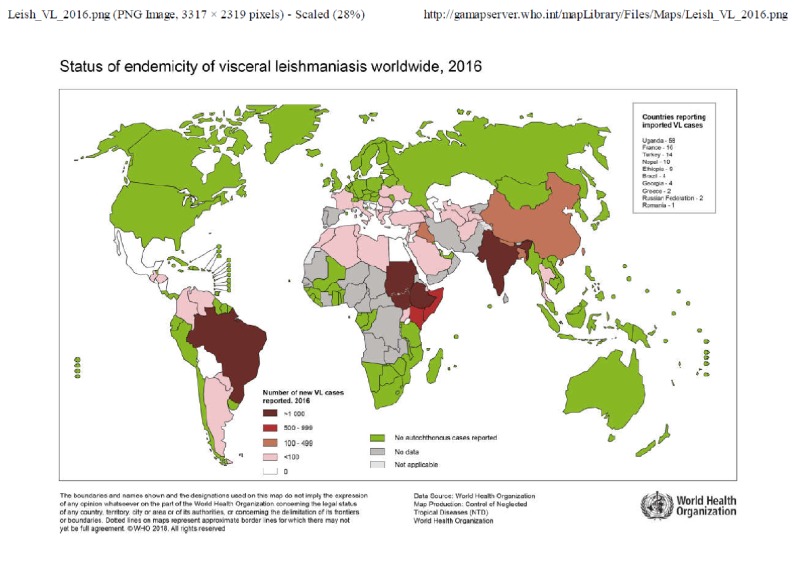
Status of visceral leishmaniasis worldwide 2016 [13].

The characteristics of the parasite and sandflies species, the local ecological characteristics of the transmission sites, current and past exposure of the human population to the parasite, and human behavior determine Leishmaniasis epidemiology [1]. HVL is rarely described in West Africa. An alternate hypothesis is that HVL could be misdiagnosed or not reported by health workers because common signs of VL such as splenomegaly and fever overlap with malaria, which is endemic in the region. In addition, HVL endemicity in West Africa is not well established, as illustrated by the status of visceral leishmaniasis in West Africa in 2016. At that time, no data were reported (see Figure 2), implying that the HVL cases reported years ago were either imported or sporadic.

One article from Senegal described risk factors associated with HVL using data from a Western blot seroprevalence study, including age over 40 years and presence of infected dogs in the household and Nebedaye trees (*Moringa oleifera*) in the area [20]. In addition, genetic risk factors may also modify VL risk in West Africa, though these factors are not yet determined.

In the district of Mont-Rolland, Thiès, Senegal, HVL Western blot seroprevalence from a survey conducted in the entire population was 23% (73/315); but symptomatic cases were not diagnosed [20]. Recently* L. infantum-*related cutaneous leishmaniasis was described in an HIV-infected child in Senegal [21]. This case of human* L. infantum*-cutaneous leishmaniasis [21], CVL reported in the Mont Roland area [5], and the possibility of a unique and recent introduction of Mediterranean* L. infantum* strain in Senegal [22] provide evidence that* L. infantum* is present in the West African region.

VL indirect hemagglutination test (IHA) screening using* L. donovani* antigen performed in 96 Malian patients with splenomegaly and leucopenia found 10 positive patients [23].

Since 1948, around 168 cases of HVL have been identified in Niger. Among these, 12 had confirmatory testing by microscopy [12, 24] and 9 were diagnosed using serology [25]. The case fatality rate in a case series of 6 cases was 33% [12]. In Niger, HVL seroprevalence was 2.2% in 90 school aged children; HVL incidence was 94 cases for a population of several hundred thousand inhabitants from 1948 to 1991 [12] and 2 cases were also found among 520 soldiers from 1992 to 1995 who had a short period stay in the same area of Tin-Galène, north of Aïr, and close to the Algerian border [25].

HVL exists in Algerian Sahara and in south of Algeria, as several cases of HVL have been observed at Hoggar (Central Sahara), and in the regions of Tamanrasset and Djanet [26, 27].

HVL incidence at the University Hospital Center of Cocody in Abidjan, Ivory Coast, was 0.56% (3/528) over a one-year follow-up period (2001-2002) [11]. We estimated a case fatality rate of 62.5% (5/8 cases) based on reports from Ivory Coast [10, 11, 28], a rate that may underestimate the death rate as some studies may not have reported clinical outcomes.

### 4.2. Vectors

In Ivory Coast, HVL vectors are not described in the literature.* Phlebotomus orientalis* and* P. alexandri* confirmed vectors, respectively, of* L. donovani* and* L. infantum*, have not been identified in Mali, Senegal, and Burkina Faso [29–31]. The absence of vectors does not necessarily exclude local* L. donovani* transmission, as* Phlebotomus (Anaphlebotomus) rodhaini* (Parrot, 1930), previously described as a possible vector of* L. donovani* [32] has been found in Mali and Senegal [29, 31]. In Mont-Rolland district, Thiès, Senegal, where canine and human VL infection has been well documented,* Sergentomyia dubia*,* Se. schwetzi*, and* Se. magna* have been incriminated as vectors of* L. infantum* [31].

In Niger, vectors of* L. donovani*, including* P. orientalis* and* P. alexandri*, have been reported [33–35]. These findings suggest that* L. donovani* transmission is possible in northern Niger, and HVL cases reported from this country are likely related to* L. donovani*. No cases of CVL have been reported in northern Niger, and the anthropophilic* L. donovani* vectors were found in the same area where HVL cases were reported.

*L. donovani* and* L. infantum *transmission is possible in West Africa where the reservoirs and vectors (*P. rodhaini*,* Se. dubia*,* Se. schwetzi*, and* Se. magna*) are endemic [29–31]. A* P. rodhaini* female has been found infected by* L. donovani* [32] and the life metacyclic* L. infantum* has been identified in* Se dubia* and* Se schwetzi* females [36]. These findings highlight the possible role of these sandflies species in visceral leishmaniasis transmission among animals and between humans and animals. Future experimental studies are needed to determine the proven role of these sandflies in this area.

### 4.3. Reservoirs

Dogs were confirmed reservoirs of HVL in West Africa [7, 14]. Carnivorous such as* Vulpes pallida* and* Genetta genetta senegalensis* were reported as possible wild reservoirs in Senegal [14]. The role of these wild animals as primary reservoirs needs to be investigated. In Burkina Faso,* L. infantum* DNA has been identified in 3 out of 5 symptomatic dogs using serology testing [6].

A Nigerian seroprevalence survey indicated that VL is transmitted among domestic dogs. An increased seroprevalence 14.63% (6/41 dogs) was recorded in Kara state [37]. In Mont Rolland district, Thiès, Senegal, canine seroprevalence was much greater at 44.9% (92/205 dogs) [5]. One dog was also incriminated as a VL reservoir in The Gambia [7]. In Ivory Coast and Niger, CVL has not been documented. Serological tests to detect VL infection in dogs in Bamako, Mali, were negative (Veterinary Central laboratory, unpublished data).

In addition to dogs, the role of mammals such as rodents as reservoirs of VL deserves further investigation in West African context. Recent studies implicated rodents as possible vectors of* L. infantum* in Morocco [38]. Elsewhere the crab-eating fox* Cerdocyon thous*, opossums* Didelphis spp.*, domestic cat* Felis cattus*, black rat* Rattus rattus*, and humans can infect sandflies. The role of these hosts as primary or secondary reservoirs requires further investigation, including xenodiagnosis studies [39].

VL reported in West Africa is mostly a zoonosis. HVL and CVL are widely distributed in West Africa, with eight countries affected (see Figure 1). In 2017, the WHO classified Senegal, Mauritania, Ivory Coast, and Niger as endemic countries whereas The Gambia and Nigeria were cited as countries with previously reported cases of HVL [13]. VL-*Leishmania* species identification in humans and dogs in West Africa is essential to guide development of effective VL prevention strategies.* L. infantum* could be the main parasite involved in West African VL. Although* L. donovani* has been cited also as causative parasite of HVL in West Africa [40, 41], it has not yet been detected in humans or animals in the region.

Although HVL are no longer described from old foci such as Ivory Coast, The Gambia, and Niger [7, 10, 11, 25, 42], VL should be investigated as both vectors and reservoirs are present, making possible reemergence in West Africa.

### 4.4. Clinical Features

In Niger, from 1948 to 1991 three HVL cases were confirmed by microscopy. From 1992 to 1995 six cases were described; common signs and symptoms at presentation included fever (n=4, jaundice (n=3), splenomegaly (n=1), weight loss (n=3), anorexia (n=4), and overall poor health status (n=5). Brucellosis and suspected liver cancer were associated with two of these HVL cases [12]. From 1985 to 1991, two cases have been diagnosed by microscopy, including one case in 1988 confirmed by microscopy out of 9 patients positive by immunofluorescence testing [25].

In Ivory Coast, Eholié* et al*. reported three cases of HVL. Clinical features at presentation included a combination of splenomegaly, overall poor health status, multiple lymphadenopathies, anarchic fever, anemia, superficial lymphadenopathy, and pleurisy. Predisposing factors that may have increased HVL risk include chronic diseases such as leukemia, Burkitt lymphoma, malaria, anemia, and malnutrition. Serological tests of VL and HIV were negative. Diagnosis of HVL was made using microscopic examination of spleen aspirate; two of these three patients died [10].

Kaoussi et al. described VL in Ivory Coast in three patients with potential predisposing conditions. One patient had a long course of systemic steroid therapy (2 months), and HIV infection was present in the two patients. Two of these three patients died [11].

Two cases were reported by Kacou et al. in Ivory Coast. The first patient had hepatomegaly and lymphadenopathy and had recently undergone 8-week systemic steroid therapy for medullar aplasia. He spent one month in a* Leishmania infantum *endemic area (Dakar, Senegal) and was positive for HIV-1. The second patient was HIV-negative and had chronic diarrhea, recurrent genital herpes, lymphadenopathy, hepatomegaly, and conjunctival pallor. Both patients had fever at presentation and positive lymph node aspirate by microscopy. One of these two patients died [28]. Unfortunately,* Leishmania* species infecting these two cases were not identified (Table 1).

In total, 17 cases of confirmed HVL have been identified, including four cases of coinfection VL/HIV, three in Ivory Coast, and one in Guinea Bissau [11, 28, 43]. HVL was seen more often in adult patients with concomitant chronic diseases other than in HIV. Immunosuppression with <200 CD4 cells/mm^3^ is a risk factor for HVL, and it is VL that often kills the patient.

### 4.5. Diagnosis and Treatment

Diagnosis is sometimes a challenge as HVL mimics viral infections, chronic malaria, leukemia, and autoimmune diseases. The presence of pancytopenia, a condition that evokes HVL [44], should lead to a diagnostic test for HVL in West African patients. Laboratory assessment should not solely rely on microscopy, as this method, despite its good specificity, has limited sensitivity. Serological testing should be used where available, including immunofluorescent assay (IFA), enzyme-linked immunosorbent assay (ELISA), immunoblotting, and Rapid Diagnosis Tests (RDTs). Microscopy may have reduced sensitivity but it is highly specific. Serologic methods are considered more sensitive despite poor results in immunocompromised patients, except immunoblotting that gives excellent results even in immunocompromised subjects. Use of molecular assays has demonstrated increased sensitivity and specificity for VL diagnosis [45]. Real-time PCR is essential for parasite identification, quantification of parasite DNA, and monitoring response to HVL and CVL treatment [46, 47]. PCR and quantitative RTPCR tests, though requiring significant infrastructure and cost investment, are thus preferred for HVL and CVL diagnosis in suspected cases and for mapping the parasite species distribution in West Africa.

Little data exists to guide VL treatment in West Africa. WHO does not have specific recommendations for VL treatment in West Africa, but for East Africa it recommends a 17-day combination therapy with sodium stibogluconate (SSG) plus paromomycin as first line therapy for VL caused by* L. donovani* [48] and 3-6 days of intravenous liposomal amphotericin B for VL caused by* L. infantum* in endemic areas [49]. Amphotericin B and meglumine antimoniate have been used as monotherapy to treat patients in Ivory Coast, Niger [10–12]. Both drugs have been used to treat one patient in Ivory Coast [10]. In Sudan and Kenya, three treatment regimens for HVL have been tested: liposomal amphotericin B plus a 10-day course single dose of SSG, liposomal amphotericin B plus 10 days of miltefosine, and miltefosine alone, showing 87%, 77%, and 72% efficacy, respectively [48]. In Brazil, N-methylglucamine antimoniate or amphotericin B deoxycholate has shown similar efficacy [50].

The clinical presentation and prognosis of VL-HIV coinfection are not well described in the West African context compared to other areas. In the Mediterranean region, VL-HIV coinfection is characterized by decreased VL cure rates, and increased drug toxicity, increased relapse and mortality rates when compared with HIV-negative VL patients [51]. VL healed with CD4 uptake in subjects coinfected with HIV. People with post-kala-azar dermal leishmaniasis are considered a potential source of VL infection [1]. In West Africa no cases of post-kala-azar dermal leishmaniasis have been described.

## 5. Conclusion

Our review suggests that VL is present in West Africa, albeit at a much lower level than in East Africa.* L. infantum* is likely the main species involved in human infection. The transmission of* L. infantum* to humans is globally established and the life cycle of* L. infantum* is known. But, HVL epidemiology in West Africa, including reservoirs, seasonality, high risk groups, and burden of disease is poorly understood. Future research is needed to understand why symptomatic VL is so rarely reported in humans. In areas where HVL have been reported with no documentation of reservoirs, studies of dogs and rodents are needed to identify parasite carriers so that effective public health interventions can be designed. Future studies will help to understand the interaction between the parasite, hosts, and vectors in areas of West Africa previously endemic for VL.

## Figures and Tables

**Table 1:** VL cases, vectors, and animals in West Africa.

Country/location	Cases				Vectors	Animals
	Number	Date	Age (years)	sex		
Mali	0	no data				
Senegal	0	no data			Sergentomyia sp.	Domestic dogs
Niger	94	1948-1991			*P. orientalis, *	
	1	Sep-92	23			
	1	Mar-93	24		*P. alexandri *	
	1	Mar-93	30			
	1	Mar-93	36			
	1	Apr-93	37			
	1	Dec-94	25			
Nigeria	1	2005				
	2	2007				
	57	2012				Domestic dogs
The Gambia	1	1949	15	Male		
	1	1980	6	Female		
	1	1982	6	Female		
Burkina Faso	1	1978		Male		Domestic dogs
Togo	1	1994				
Guinea	0	no data				
Guinea-Bissau	1	1990				
Liberia	0	no data				
Ghana	0	no data				
Mauritania	0	no data				
Cote d'Ivoire	1	9-Jun-04	31	Female		
	1	14-Sep-04	65	Female		
	1	25-Apr-05	5			
	1	2004	37	Female		
	1	2004	26	Female		
Total	171					

## References

[1] WHO Leishmaniasis Fact Sheet Update March 2018. http://origin.who.int/mediacentre/factsheets/fs375/en/.

[2] Desjeux P., Jannin J., Bern C. (2012). Leishmaniasis worldwide and global estimates of its incidence. *PLoS ONE*.

[3] WHO Leishmania Endemic Countries, 2017a. http://apps.who.int/gho/athena/data/GHO/NTD_LEISHVEND?filter=COUNTRY:∗&;format=xml&;profile=excel.

[4] Davidson R., Croft S. (1992). Visceral leishmaniasis in Africa. *Africa Health*.

[5] Faye B., Bañuls A., Bucheton B. (2010). Canine visceral leishmaniasis caused by Leishmania infantum in Senegal: risk of emergence in humans?. *Microbes and Infection*.

[6] Sangaré I., Djibougou A., Yaméogo B. K. (2016). First detection of leishmania infantum in domestic dogs from burkina faso (West Africa). *Research Journal of Parasitology*.

[7] Desjeux P., Bryan J. H., Martin-Saxton P. (1983). Leishmaniasis in the gambia. 2. a study of possible vectors and animal reservoirs, with the first report of a case of canine leishmaniasis in the gambia. *Transactions of the Royal Society of Tropical Medicine and Hygiene*.

[8] Olmer J. (1972). Adult kala-azar observed on the ivory coast. *Bulletin De l'Academie Nationale De Medecine*.

[9] de Campos E. P., Amedomé A. A., Kpodzro K. (1979). Kala-azar in Togo, West Africa. presentation of a clinical case. *Revista Do Instituto de Medicina Tropical de Sao Paulo*.

[10] Eholié S. P., Tanon A. K., Folquet-Amorissani M. (2008). Three new cases of visceral leishmaniasis in Côte dIvoire. *Bulletin de La Societe de Pathologie Exotique (1990)*.

[11] Kouassi B., Horo K., Achi V. H. (2005). Three cases of visceral leishmaniasis in abidjan, Cote d’Ivoire. *Medecine Tropicale: Revue Du Corps de Sante Colonial*.

[12] Djidingar D., Chippaux J. P., Gragnic G., Tchani O., Meynard D., Julvez J. (1990). Visceral leishmaniasis in Niger: six new parasitologically confirmed cases. *Bulletin de La Societe de Pathologie Exotique*.

[13] WHO Weekly Epidemiological Record. http://www.who.int/leishmaniasis/resources/REH_38_TABLEAU_S1_S2_Version_finale.pdf?ua=1.

[14] Desjeux P. (1991). *Information Sur l’épidémiologie Des Leishmanioses et La Lutte Contre Les Maladies Par Pays Ou Territoire*.

[15] Conteh S., Desjeux P. (1983). Leishmaniasis in The Gambia. I. A case of cutaneous leishmaniasis and a case of visceral leishmaniasis. *Transactions of the Royal Society of Tropical Medicine and Hygiene*.

[16] Greenwood B., Ajdukiewicz A., Conteh S., Hagan P., Mabey D., Panton L. (1984). Leishmaniasis in the gambia. 3. is its incidence increasing?. *Transactions of the Royal Society of Tropical Medicine and Hygiene*.

[17] Andre L. J., Sirol J., Le Vourch C., Labegorre J., Cochevelou D. (1978). Sudanese kala-azar in West Africa (Authors Transl). *Medecine Tropicale: Revue Du Corps de Sante Colonial*.

[18] Kamara B. L., Safianova V. M., Goncharov D. B., Emelianova L. P. (1992). The serological examination of the population for leishmaniasis and the detection of leishmania in rodents in the Republic of Guinea. *Meditsinskaia Parazitologiia i Parazitarnye Bolezni*.

[19] Reithinger R., Brooker S., Kolaczinski J. H. (2007). Visceral leishmaniasis in eastern africa – current status. *Transactions of the Royal Society of Tropical Medicine and Hygiene*.

[20] Faye B., Bucheton B., Bañuls A. L. (2011). Seroprevalence of Leishmania infantum in a rural area of Senegal: analysis of risk factors involved in transmission to humans. *Transactions of the Royal Society of Tropical Medicine and Hygiene*.

[21] Diatta B., Diallo M., Diadie S. (2016). Cutaneous leishmaniasis due to leishmania infantum associated with HIV. *Annales de Dermatologie et de Vénéréologie*.

[22] Cassan C., Dione M. M., Dereure J. (2016). First insights into the genetic diversity and origin of leishmania infantum in mont rolland (thiès region, Senegal). *Microbes and Infection*.

[23] Blanchot M., Slavov R., Rhaly A. A., Digoutte P. (1984). Attempt at evaluating the validity of immunochemical methods of detecting leishmaniasis. *Dakar Medical*.

[24] Doury P. (1989). A New focus of kala-azar in Aïr (Niger). *Medecine Tropicale: Revue Du Corps de Sante Colonial*.

[25] Laporte P., Decroix Y., Chevauchee P. (1988). A focus of kala-azar in aïr (Niger): first confirmed autochthonous Nigerian case. *Medecine Tropicale: Revue Du Corps de Sante Colonial*.

[26] Doury P. (1957). Three new autochthonous cases of general leishmaniasis (Mediterranean kala-azar) observed at Hoggar (Central Sahara). *Archives de l'Institut Pasteur d'Algérie Institut Pasteur d'Algérie*.

[27] Doury P. (1956). Two autochthonous cases of general leishmaniasis (mediterranean kala-azar) observed at Hoggar (central Sahara). *Archives de l'Institut Pasteur d'Algérie Institut Pasteur d'Algérie*.

[28] Kacou E. S., Ouhon J. (2000). *Leishmaniose Viscérale: À Propos de 2 Cas Colligés Au Service de Pneumophtisiologie Du CHU de Cocody: Observations et Revue de La Littérature*.

[29] Berdjane-Brouk Z., Koné A. K., Djimdé A. A. (2012). First detection of leishmania major DNA in sergentomyia (Spelaeomyia) darlingi from cutaneous leishmaniasis foci in mali. *PLoS ONE*.

[30] Sangare I., Gantier J., Koalaga G., Deniau M., Ouari A., Guiguemdé R. (2009). Sandflies of the south part of ouagadougou city, Burkina Faso. *Parasite*.

[31] Senghor M. W., Faye M. N., Faye B. (2011). Ecology of phlebotomine sand flies in the rural community of Mont Rolland (Thiès region, Senegal): area of transmission of canine leishmaniasis. *PLoS ONE*.

[32] Elnaiem D.-E. A., Hassan H. K., Osman O. F., Maingon R. D. C., Killick-Kendrick R., Ward R. D. (2011). A possible role for phlebotomus (anaphlebotomus) rodhaini (parrot, 1930) in transmission of leishmania donovani. *Parasites & Vectors*.

[33] Parrot L., Doury P. (1955). Notes on phlebotomes. LXVIII. the new hoggar phlebotomes. *Archives de l’Institut Pasteur d’Algerie. Institut Pasteur d’Algerie*.

[34] Abonnec E., Dyemkouma A., Hamon J. (1964). On the presence of phlebotomus (phlebotomus) orientalis parrot, 1936, in the republic of Niger. *Bulletin de La Societe de Pathologie Exotique et de Ses Filiales*.

[35] Le Pont F., Robert V., Vattier-Bernard G., Rispail P., Jarry D. (1993). Notes on the phlebotomus of aïr (Niger). *Bulletin de la Société de Pathologie Exotique (1990)*.

[36] Senghor M. W., Niang A. A., Depaquit J. (2016). Transmission of leishmania infantum in the canine leishmaniasis focus of mont-rolland, senegal: ecological, parasitological and molecular evidence for a possible role of sergentomyia sand flies. *PLOS Neglected Tropical Diseases*.

[37] Adediran O. A., Kolapo T. U., Uwalaka E. C. (2016). Seroprevalence of canine leishmaniasis in Kwara, Oyo and Ogun states of Nigeria. *Journal of Parasitic Diseases*.

[38] Echchakery M., Chicharro C., Boussaa S. (2017). Molecular detection of Leishmania infantum and Leishmania tropica in rodent species from endemic cutaneous leishmaniasis areas in Morocco. *Parasites & Vectors*.

[39] Quinnell R. J., Courtenay O. (2009). Transmission, reservoir hosts and control of zoonotic visceral leishmaniasis. *Parasitology*.

[40] Kimutai A., Ngure P. K., Tonui W. K., Gicheru M. M., Nyamwamu L. B. (2010). Leishmaniasis in northern and western Africa: a review. *African Journal of Infectious Diseases*.

[41] Boakye D. A., Wilson M., Kweku M. (2005). A review of leishmaniasis in West Africa. *Ghana Medical Journal*.

[42] Gaultier Y., Peccarère J., Deyeloux M. (1989). Visceral leishmaniasis in Niger. *Transactions of the Royal Society of Tropical Medicine and Hygiene*.

[43] Desjeux P. (1995). Leishmania/HIV co-infections. *Africa Health*.

[44] Koster K.-L., Laws H.-J., Troeger A., Meisel R., Borkhardt A., Oommen P. T. (2015). Visceral leishmaniasis as a possible reason for pancytopenia. *Frontiers in Pediatrics*.

[45] Srivastava P., Mehrotra S., Tiwary P., Chakravarty J., Sundar S. (2011). Diagnosis of indian visceral leishmaniasis by nucleic acid detection using PCR. *PLoS ONE*.

[46] Hossain F., Ghosh P., Khan M. A. (2017). Real-time PCR in detection and quantitation of Leishmania donovani for the diagnosis of Visceral Leishmaniasis patients and the monitoring of their response to treatment. *PLoS ONE*.

[47] Rampazzo R. d., Solcà M. d., Santos L. C. (2017). A ready-to-use duplex qPCR to detect Leishmania infantum DNA in naturally infected dogs. *Veterinary Parasitology*.

[48] Wasunna M., Njenga S., Balasegaram M. (2016). Efficacy and safety of ambisome in combination with sodium stibogluconate or miltefosine and miltefosine monotherapy for african visceral leishmaniasis: phase II randomized trial. *PLOS Neglected Tropical Diseases*.

[49] WHO Box 1. Recommended Treatment Regimens for Visceral Leishmaniasis, Ranked by Preference1. https://www.who.int/leishmaniasis/research/978924129496_pp67_71.pdf?ua=1.

[50] Borges M. M., da Silva Pranchevicius M. C., Noronha E. F., Romero G. A. S., Carranza-Tamayo C. O. (2017). Efficacy and safety of amphotericin B deoxycholate versus N-methylglucamine antimoniate in pediatric visceral leishmaniasis: an open-label, randomized, and controlled pilot trial in Brazil. *Journal of the Brazilian Society of Tropical Medicine*.

[51] Monge-Maillo B., Norman F. F., Cruz I., Alvar J., López-Vélez R. (2014). Visceral leishmaniasis and HIV coinfection in the mediterranean region. *PLOS Neglected Tropical Diseases*.

